# Supplementary figures and images for: Comparative study of nasal cavity drug delivery efficiency with different nozzles in a 3D printed model
Source: PeerJ. 2024 Apr 10;12:e17227. doi: 10.7717/peerj.17227 (PMC11015827; doi:10.7717/peerj.17227)

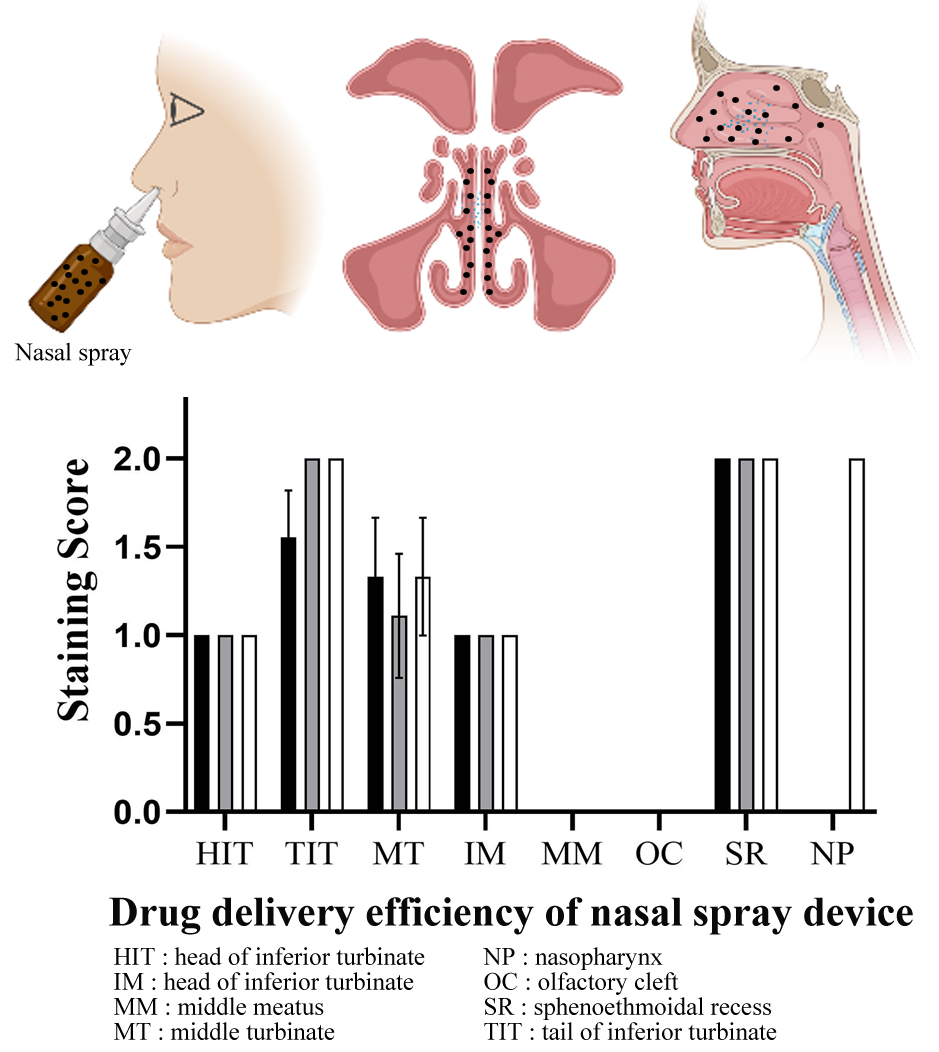

Supplement: Supplemental Information 1 — The upper panels simulate the overall distribution of nasal sprays in the nasal cavity. Because of the complexity of anatomy on the lateral nasal wall, the distribution of nasal sprays on the lateral nasal wall was assessed using a scoring system. The histogram at the bottom shows the scores for different anatomical regions. Created with BioRender.com. [file peerj-12-17227-s001.jpg]
